# Clinical decision support for potentially sedating drugs in palliative care: a mixed-methods pilot study

**DOI:** 10.1186/s12904-026-02346-z

**Published:** 2026-10-01

**Authors:** Sabine H. Krauss, Beatrice Odierna, Stefanie Kolmhuber-Seibold, Constanze Rémi, Claudia Bausewein, Carsten Klein, Christoph Ostgathe, Alexander Kremling, Jan Schildmann, Eva Schildmann

**Affiliations:** 1 Department of Palliative Medicine, LMU Medizin, LMU University Hospital, Ludwig-Maximilians-Universität München, Marchioninistr. 15, Munich, D-81377 Germany; 2 Department of Palliative Medicine, CCC Erlangen – EMN, University Hospital Erlangen, Friedrich-Alexander-Universität Erlangen-Nürnberg (FAU), Krankenhausstraße 12, Erlangen, D-91054 Germany; 3 Institute for History and Ethics of Medicine, Interdisciplinary Centre for Health Sciences, Martin Luther University Halle-Wittenberg, Magdeburger Straße 8, Halle (Saale), D-06112 Germany; 4 Palliative Medicine, Faculty of Medicine, University of Augsburg, Stenglinstraße 2, Augsburg, D-86156 Germany

**Keywords:** Palliative care, Suffering, Administration and dosage, Sedatives, Palliative sedation, Deep sedation, Light sedation, Clinical decision support, Mixed-methods

## Abstract

**Background:**

Within the framework of the project iSedPall, a Clinical Decision Support Tool (CDST) was developed and piloted to address uncertainties in the use of potentially sedating drugs commonly administered in palliative care. The so-called ‘warning list’ contains evidence- and expert consensus-based data. This study aimed to test its feasibility of implementation, appropriateness, and acceptability.

**Methods:**

Mixed-methods pilot study including data from four pilot centres (two inpatient palliative care units, two palliative home care teams): a retrospective cohort study by chart review, an online survey, and focus groups (one focus group per pilot centre and two setting-specific focus groups). Quantitative data was analysed via descriptive statistics and content analysis was used for the focus groups.

**Results:**

Retrospective cohort study (*n* = 1,189 patients): The frequency of the use of the warning list depended on the patient group. The list was used most frequently for patients in whom sedation occurred as an adverse drug reaction. Online survey: 13/33 respondents used the list. Eight planned to continue using the warning list after the end of the project. Focus groups (six focus groups with overall *n* = 22 professionals): Generally, the aim of the warning list was well understood. The benefits were seen primarily in terms of education and background information. Participants also suggested the availability in electronic form. A potential risk is that the warning list could be misinterpreted as dose recommendations for sedation.

**Conclusions:**

The warning list seems to be appropriate, acceptable, and feasible to be implemented for professionals, according to pilot evidence from four centres. The mixed-methods approach proved valuable in carefully exploring the risks alongside the benefits. Future research should be conducted in more generalisable settings, take a closer look at different professional groups as well as settings, and further explore the various types of potential use of the CDST. Implementation in electronic form should be explored carefully, reflecting positive and negative consequences on daily routines.

**Trial registration:**

The study was registered in the German Clinical Trials Register (DRKS-ID: DRKS00027241; Date of registration: 10/12/2021; https://drks.de/search/en/trial/DRKS00027241).

**Supplementary Information:**

The online version contains supplementary material available at https://doi.org/10.1186/s12904-026-02346-z.

## Introduction / background

Potentially sedating drugs are frequently used in palliative care, typically aiming to improve the quality of life of severely ill patients. However, in clinical practice, there are uncertainties in their use when it comes to recognizing sedative effects – and therefore uncertainties exist regarding the benefits and harms for the patients: it is often challenging to distinguish between reduced consciousness due to illness and sedation caused by medication. It can also be difficult to recognize when the use of potentially sedating drugs such as benzodiazepines for anxiolysis starts to cause sedation [1]. However, recognizing, or ideally anticipating, sedation is crucial for decision making regarding dose reduction, substance change, or intentional sedation to relieve suffering, as well as providing adequate information and obtaining informed consent [2–6]. As part of the research project iSedPall (“Development and piloting of a multimodal intervention for the use of sedating drugs in specialist palliative care”) [7], a tool, the ‘warning list’ (Additional file 1 – Warning list), was developed to address these issues with high practical relevance in the use of sedative drugs [8–10]. Recommendations on the use of sedative drugs in palliative care had been approved by an expert consensus process in the previous project, SedPall [11]. iSedPall aimed to develop and pilot practical tools to support the transfer of these recommendations into practice. The warning list is part of this set of tools (‘toolbox’) [8, 9, 12, 13].

The warning list contains evidence-based, expert consensus-based cut-off values for selected potentially sedating drugs commonly used in palliative care, each for a defined ‘standard patient’: cut-off dosing intervals for continuous clinical effects and cut-off-doses for (a) light or (b) deep sedation (see Additional file 1 – Warning list). The list is intended to support the evaluation in two situations: (1) whether a given or planned dose of a potentially sedating drug, not prescribed with the aim of sedation, might have a sedative effect; (2) whether an observed reduced consciousness was probably (partially) caused by the medication [10]. As indicated on the list (Additional file 1 – Warning list), it is aimed to raise awareness whether sedative effects can be expected and to support the respective judgement. It therefore supports professionals in palliative care primarily by providing information, namely a compilation of relevant potentially sedating drugs and critical doses and dosing intervals. With this information, cases of sedation should be more easily foreseen or identified and further decisions made (e.g. information about potential sedative side effects, change of medication or information of patients and/or relatives about an intentional sedation) – depending on the (presumed) will of the patient and the improvement of his or her quality of life.

The warning list can be seen as a paper version of a clinical decision support tool (CDST). Non-electronic CDSTs can consist of forms, rules and methods based on decision trees or schemes [14]. The aims of such tools – either as paper or electronic version – are broad, e.g. to provide knowledge and give recommendations [15], to identify and select patients [16–21], or to improve confidence [22]. CDSTs can improve the practice of professionals and therefore can influence the treatment of patients in a positive way. Such tools are developed for specific questions or situations on the basis of data and evidence. They are suitable for making changes to daily clinical practice and informing both care providers and patients [23–25], and are gaining relevance for palliative care [26–28].

The warning list was mainly developed based on a literature review and a group Delphi process [10]. In addition, think-aloud cognitive interviews and a constant exchange between research and practice within a pre-pilot phase helped to refine the tool, details are reported elsewhere [8, 13, 29]. The list was distributed to all four pilot centres as part of the set of tools (‘toolbox’), comprising meetings of researchers with members from the pilot centres and self-training material for all members of staff [9, 13]. The overall aim of the study reported here was to test the feasibility of implementation, appropriateness, and acceptability of the warning list.

## Methods

### Study design, setting, and participants

As the methods are reported elsewhere in more detail [8, 13, 29], we only provide summarized information here. The mixed-methods pilot study was conducted in two palliative care units and two palliative care home care teams in Germany. It comprised a retrospective cohort study by chart review, an online survey among the participating team members, and six focus groups (one per team and additionally one per each setting; with physicians, nurses, and other professionals such as study nurses and a psychologist).

### Data collection

The retrospective cohort study by chart review included all patients cared for in the four pilot centres from May 2023 till January 2024 [29]. Study nurses extracted the following data from patient records: (i) whether a patient had received potentially sedating drugs and which drugs; (ii) whether a cut-off-value (for light/deep sedation) provided in the warning list was reached for the respective drug; (iii) whether the warning list had been used; (iv) whether the patient died or was discharged. The potentially sedating drugs which were documented were those included in the warning list (diazepam, lorazepam, midazolam, levomepromazine/methotrimeprazine, haloperidol, melperone, pipamperone, propofol, dexmedetomidine, trazodone). Additionally, phenobarbital was included, as this drug was originally intended to be part of the tool [10]. Professionals from the pilot centres were asked to state if they had used the warning list, in combination with questions regarding various other data needed for the retrospective cohort study, on an additional form attached to the patient record. The exact way how the warning list was used during the course of a patient’s treatment was not further specified. Data were transmitted in anonymised form to the research team via an Excel file. In addition, the responsible physicians were asked to allocate each patient to a group category: group 1 – no potentially sedating drugs (as listed on the ‘warning list’) given to the patient; group 2 – no sedation intended and no sedation occurred; group 3 – intentional sedation to relieve suffering; group 4 – sedation considered but not realized; group 5 – sedation as an adverse drug reaction (and adjusting the administered drug is not possible) [29]. Sedation and depth of sedation was defined in line with the RASS-PAL scale [2, 10, 30].

The online survey was conducted via the online survey tool Unipark [31]. Around *n* = 100 staff members from the four pilot centres were invited to participate. The questionnaire was distributed via e-mail by members of the pilot centres. Comprehensive details about the conduct and the participants of the online survey are reported elsewhere [9]. The questionnaire comprised two questions regarding the warning list, one asking whether the list was used (without specifying the kind of use), and the other whether the participant intended to use it after the end of the project. The online survey was conducted from September 2023 till October 2023. For organisational reasons, especially because it should take place before the focus groups, this date was a few months before the end of the pilot phase.

In total, six focus groups were conducted: one with each pilot centre and two additional ones per setting (palliative care units and home care teams). Senior physicians and nurse managers of all four pilot centres were invited to participate and were asked to invite further members of their team. The focus groups took place from October 2023 to January 2024. The interview guide was informed by preliminary results from the online survey and the Consolidated Framework for Implementation Research (CFIR) [32, 33]. It focussed on the issues of general acceptance and relevance of the set of tools, its user-friendliness and need of adaption, facilitators and barriers regarding the use of the tools, and unexpected consequences. It also contained a follow-up question addressing explicitly the drug-related warning list.

### Analysis

The retrospective cohort study and the online survey were analysed using IBM SPSS Statistics 28.0 [34]. Descriptive statistics were performed for the variables reported here.

The data from the focus groups were analysed in accordance with qualitative content analysis as described by Schreier [35]. Categories were derived in a partly deductive and partly inductive way. MAXQDA version 2022 was used to support the process [36].

## Results

### Retrospective cohort study

The cohort study by chart review included *n* = 1,189 patients, aged between 27 and 100 years (M = 75, SD = 12.7). 46% (*n* = 547) were female, 69.1% (*n* = 822) had a cancer diagnosis. Approximately two-thirds (*n* = 804/67.6%) of the patients received at least one potentially sedating drug included in the warning list (an overall description has been reported elsewhere) [29].

Seven of the eleven drugs named in the warning list were administered to patients. Lorazepam was used most frequently, followed by levomepromazine and midazolam. Phenobarbital, propofol, dexmedetomidine, and trazodone were not used. Notable variations were observed between the different drugs regarding the reached cut-off values for light or deep sedation. While the cut-off-value for light sedation to be expected was reached with each of the drugs (for 0.1 to 13% of patients), the cut-off-value for deep sedation to be expected was only reached for three drugs (lorazepam, midazolam, levomepromazine; for 0.3 to 4.8% of patients receiving these drugs). Midazolam was the drug for which both cut-off values were reached most frequently (see Table 1).

**Table 1:** Potentially sedating drugs given to patients and cut-off-values reached (Total number of patients *n* = 1,189)

Drug with potentially sedative effect	Number of patients who received the drug (% of total number of patients)	Number of patients with reached cut-off-value for light sedation to be expected with specific drug (% of total number of patients)	Number of patients with reached cut-off-value for deep sedation to be expected with specific drug (% of total number of patients)
Diazepam	2 (0.2%)	1 (0.1%)	0
Lorazepam	515 (43.3%)	16 (1.3%)	4 (0.3%)
Midazolam	252 (21.2%)	154 (13.0%)	57 (4.8%)
Levomepromazine (Methotrimeprazine)	313 (26.3%)	28 (2.4%)	6 (0.5%)
Haloperidol	112 (9.4%)	1 (0.1%)	0
Melperone	24 (2.0%)	2 (0.2%)	0
Pipamperone	119 (10.0%)	37 (3.1%)	0
No sedating drug from list administered	385 (32.4%)	-	-

Over half of all patients (*n* = 699/58,8%) received potentially sedating drugs without documentation of sedation: either “no sedation was intended and no sedation occurred” (group 2, see Table 2) or “sedation was considered but not realized” (group 4, see Table 2). Over one eighth of the patients in group 2 and over half of the patients in group 4 reached the cut-off-value for “light sedation to be expected”. Fewer, but some patients of both groups reached the cut-off-value for “deep sedation to be expected” (see Table 2). The two groups with patients for whom sedation was documented comprised patients with “intentional sedation to relieve suffering” (group 3, see Table 2) and patients with “sedation as an adverse drug reaction (without the possibility of adjusting the administered drug)” (group 5, see Table 2). Each of these two groups comprised under 5% of all patients (see Table 2). A high percentage of patients in the two groups (nearly 90% in group 3 and nearly 85% in group 5) reached the cut-off-value for “light sedation to be expected” whereas around one third reached the cut-off-value for “deep sedation to be expected” (see Table 2). These two groups also had the highest percentage of patients who died (see Table 2). The warning list was used in all groups with patients who received potentially sedating drugs (groups 2, 3, 4, and 5, see Table 2). The list was used most frequently in the group of patients with sedation as an adverse drug reaction (without the possibility of adjusting the administered drug) (group 5, see Table 2).

**Table 2:** Potentially sedating drugs and use of warning list per patient group (Total number of patients *n* = 1,189)

	Group 1 No potentially sedating drugs (as listed on ‘warning list’) given RASS-PAL ≥ 0 *n* = 385 (32.4% of all patients)* number (%**)	Group 2 No sedation intended and no sedation occurred RASS-PAL ≥ 0 *n* = 686 (57.7% of all patients)* number (%**)	Group 3 Intentional sedation to relieve suffering RASS-PAL < 0 *n* = 52 (4.4% of all patients)* number (%**)	Group 4 Sedation considered but not realized RASS-PAL ≥ 0 *n* = 13 (1.1% of all patients)* number (%**)	Group 5 Sedation as an adverse drug reaction (without the possibility of adjusting the administered drug) RASS-PAL < 0 *n* = 53 (4.5% of all patients)* number (%**)
Received potentially sedating drugs	0 (0%)	686 (100%)	52 (100%)	13 (100%)	53 (100%)
Reached cut-off-value for light sedation to be expected	0 (0%)	111 (16.2%)	46 (88.5%)	7 (53.8%)	45 (84.9%)
Reached cut-off-value for deep sedation to be expected	0 (0%)	25 (3.6%)	21 (40.4%)	2 (15.4%)	16 (30.2%)
Warning list was used	0 (0%)	65 (9,5%)	34 (65,4%)	6 (46.2%)	47 (88.7%)
Deceased	217 (56.4%)	537 (78.3%)	51 (98.1%)	12 (92.3%)	51 (96.2%)

*Source: Schneider et al. 2024 [29] – final results reported here are slightly different from the results reported in 2024, due to corrections from the participating pilot centres

**Of number of patients in this group

### Online survey

The online survey was completed by *n* = 33 participants (*n* = 9 physicians, *n* = 17 nurses, *n* = 7 members of other professions). Most participants (*n* = 28) answered the question regarding the use of the warning list during the pilot phase: almost half of the respondents (*n* = 13; among them *n* = 7 physicians and *n* = 4 nurses) had used the list. Fewer participants (*n* = 24) answered the question regarding a future use of the list: one third (*n* = 8; among them *n* = 4 physicians and *n* = 3 nurses) of the respondents would like to use the warning list after the end of the project.

### Focus groups

Twenty-two professionals from the four pilot centres participated in the focus groups which had a duration of approximately 70 to 110 min. The sample consisted mainly of physicians and nurses, most of them with more than five years of professional experience (see Table 3). Some (*n* = 5 physicians; *n* = 3 nurses) participated both in their own centre-specific focus group and in the corresponding setting-specific group.

**Table 3:** Sociodemographic and professional characteristics of the participants in the focus groups

	Physicians *n* = 10	Nurses *n* = 8	Study Nurses *n* = 3	Psychologists *n* = 1
Gender, *n* (%)				
Female	5 (50.0)	6 (75.0)	3 (100)	1 (100)
Age, n (%)				
16–25	0 (0)	0 (0)	1 (33.3)	0 (0)
26–35	1 (10.0)	1 (12.5)	2 (66.7)	0 (0)
36–45	2 (20.0)	4 (50.0)	0 (0)	0 (0)
46–55	4 (40.0)	2 (25.0)	0 (0)	0 (0)
>=56	3 (30.0)	1 (12.5)	0 (0)	1 (100)
Setting, n (%)				
Inpatient	6 (60.0)	4 (50.0)	3 (100)*	1 (100)
Home care	4 (40.0)	4 (50.0)	2 (66.7)*	0 (0)
Years of professional experience, n (%)				
< 1	1 (10.0)	0 (0)	2 (66.7)	0 (0)
1–4	3 (30.0)	1 (12.5)	0 (0)	1 (100)
>=5	6 (60.0)	7 (87.5)	1 (33.3)	0 (0)

*Two of the three study nurses worked in the inpatient and home care setting

The following main categories were used for the coding, which had been derived in a partly deductive and partly inductive way: acceptability of the warning list, relevance of the warning list, contexts of use of the warning list, and user-friendliness of the warning list.

The focus groups showed a high acceptability of the warning list. The aim of the warning list was generally well understood and the list received approval:

“So, our experience is that we like to use the list to see in which medication range we are.” (Senior physician, 5/55).

The findings also reflect the relevance of the list. For the participating professionals, the warning list was helpful for different purposes and in different occasions. Primarily, physicians and nurses appreciated its benefits in terms of education and background information:

“For members of staff who simply want to gain an overview.” (Nurse, 1/180).

“This list containing the drugs has awakened in us a renewed awareness that we should handle them with care. We use them in the same way and just as indicated, but we use them with more knowledge.” (Senior physician, 1/37).

The list was also used on a daily basis in case discussions. In such meetings, the list helped to check whether a cut-off value had been exceeded, to check what this meant for the patient and the team, to gain a joint view on the patient within the team, and to act conjointly:

“We have a fairly extensive list of PRN medication for the night. And retrospectively, it was always helpful to see to what extent the medication was actually utilized. Did we cross a dose limit due to the actual needs? So that we could then discuss this again and take another look. So, the warning list was GOOD for this purpose in the team meeting. And that also led to transparency and clarity for us.” (Senior physician, 5/131).

Such a renewed focus on potentially sedating drugs could lead to a further look on patients and their status as possibly already sedated patients. One team (senior physician, 6/23) narrated that – on basis of the warning list and an information provided by a nurse – they had realized that the medication given to a patient had led to a sedation and, as a consequence, they talked to the relatives and explained the situation to them.

With regard to the evaluation of the user-friendliness, there are mixed perceptions. A barrier for the use of the warning list can be that the list is seen as too simple to improve knowledge and not complex enough to represent the reality of patients, as the list refers to a “standard patient”. The list does not contain enough information to evaluate the situation of a specific patient and physicians need to use additional information and their own knowledge. Therefore, physicians emphasize their extensive experience with potentially sedating drugs and the requirement of individual assessments:

“I myself didn’t use the list […] that often, because I was happy to rely on my own skills in my busy everyday working life. […] The individual case is much more complicated. We are usually not talking about the standard patient. It is a mixed combination of different medications that work together. The health situation changes.” (Physician, 1/34).

Though the list is partially judged as not complex enough, as it operates with a standard patient, it is also seen as time-consuming and complex, as the way the doses are indicated on the list do not reflect the daily practice of all teams and professionals:

“I would have to recalculate what we give twice a day. It’s just complex and complicated.” (Physician, 2/30).

The user-friendliness might possibly be improved if the list could be implemented within the electronic system. Participants mentioned this solution without any questions or hints from the interviewer. Some participants would appreciate an electronic implementation:

“We could be supported quite a bit by the digital patient curve, which, for example, immediately indicates: according to the warning list, the midazolam dose exceeds a critical range. Sedative effects must be expected here. Plopp: Is that what you want?” (Physician, 1/182).

A potential risk is that the warning list is misinterpreted as dose recommendations for sedation, as was the case in the following quote, saying that pipamperone is not a suitable drug for sedation:

“There are drugs on it that we don’t need in a home care setting. And I think that’s more likely to be confusing when you start with sedation. […] How am I supposed to do palliative sedation with pipamperone? No chance.” (Senior physician 6/296–303).

## Discussion

The warning list piloted in this study is a CDST which aims to support clinical judgements regarding sedative effects of a range of potentially sedating drugs commonly used in palliative care. It provides evidence- and expert consensus-based information and explanatory notes for its use [10, 12]. It complements existing recommendations and guidelines regarding intentional sedation to relieve suffering or palliative sedation [3, 11, 37], by addressing unintentional sedation, a topic which has received little attention so far [10].

The retrospective cohort pilot study by chart review in four centres in Germany demonstrated that the warning list was used most frequently in the group of patients who were classified as experiencing sedation as an adverse drug reaction (without the possibility of adjusting the administered drug). As the warning list aims to support professionals in such situations by providing guidance for their judgement (if a reduction of consciousness could be caused by a drug – namely by providing drug dosages with which a sedative effect should be expected), an important objective of the list seems to have been met. Additionally, the findings from the focus groups suggest that the status of some of these patients might not have been explicitly declared as ‘sedated’ without the availability of the warning list, e.g. in case of PRN medication for the night which was discussed in a team meeting. Other situations where the list was frequently used were those in which intentional sedation to relieve suffering was either undertaken or considered. These results support the idea that the warning list provides guidance in such complex situations, including adequate labelling of the treatment as prerequisite for the application of best practice guidelines.

Professionals participating in the study in the four pilot centres seem to see an advantage in the use of the warning list in many, but not all cases: in patients receiving potentially sedating drugs without intended sedation (and no sedation occurred), the warning list was used only for one of ten patients, while over 16% of these patients reached the cut-off dose where light sedation is to be expected. Nevertheless, the results can be seen as an indication that the cut-off values on the list are clinically plausible: more than 80% of patients in the group of no intended sedation (and no sedation occurred) received doses lower than the suggested cut-off values for light sedation to be expected. This interpretation is supported by the similarly high percentage in the group of patients with intentional sedation: nearly 90% of them reached the cut-off-value for light sedation to be expected. Further work would be needed to formally validate the cut-off values.

Complementary to the results of the quantitative study, the qualitative data of this pilot study in four German centres underline that professionals see the main benefit of the warning list in providing background information and raising awareness on drugs and dose ranges potentially causing light and deep sedation. It thus can help to strengthen professionals’ confidence in using sedating drugs. Though the list also supported discussions of medication of individual patients in their daily practice, users emphasise the need for individual assessment. Accordingly, the acceptance of the list seems to be focused on occasions when new staff need to be trained: the list may help to raise awareness and to get familiar with the topic of sedative (side) effects of potentially sedating drugs. It might additionally be the starting point for judging the situation of individual patients – by providing data for a defined ‘standard patient’.

Lists are generally seen as important way to compile and provide knowledge about drugs in palliative care [38, 39]. This pilot study provides preliminary evidence of the feasibility of implementing the warning list in clinical practice. It showed that the list can provide users with relevant information in an appropriate form. The list therefore could offer a broad support to professionals and fulfils many of the advantages of CDSTs reported in the literature [15, 19, 20, 22, 23, 26]. Barriers for the use of the list result from the need to transfer the information from the list into an individual situation, adding patient-specific details. The pilot has also shown that there is a potential risk associated with the use of the list: the warning list could be misinterpreted as dose recommendations for intentional sedation. To reduce this risk of misinterpretation, dose recommendations for the start of intentional sedation to relieve suffering were developed as an additional CDST within the set of tools (see Additional file 2 – Dose recommendations) [8, 12].

Users mentioned that an electronic implementation could be more efficient in helping them in their daily routine. An electronic version could use existing data and IT infrastructure, and provide automatically the relevant information and a call for action in the exact moment where both is needed [14, 21, 40], as also suggested in the focus groups. This might change daily practice more thoroughly. At the same time, this might bring new difficulties: the literature reports that more time is needed for education and too many alarms can cause an “alert fatigue” [41]. The daily practice might also be influenced in a negative way regarding patients, e.g. if there’s more focus on the data instead of the individual patients [15, 42]. Some of these concerns are already reflected in the feedback of individual participants of the focus groups regarding the paper version: that the warning list cannot model the patient’s situation well enough and an individual approach, combined with a broad knowledge base of the users, is always needed. Nevertheless, though electronic CDSTs are rarely implemented in palliative care and their adequate use requires further general discussion, they might support medication management and care planning [26, 28]. An (additional) paper version seems to be appropriate, though. It can be used wherever no electronic devices are available or possibly not wanted, e.g. in home care settings or team meetings. This might also be helpful in the format of a pocket card. Both for the paper version as well as for electronic formats it remains crucial to emphasize what a CDST can and cannot do: its role is to support decisions, in the case of the warning list based on information regarding a hypothetical ’standard patient’. Making judgements and decisions for individual patients always remains the task of the team caring for the patient – ideally supported by the CDST, taking all individual circumstances into account.

### Strengths and limitations

The mixed-methods approach has proven to be valuable to gain knowledge about the practical use of the warning list and to explore the perspective of the users. Especially the qualitative approach, i.e. the focus groups, gave valuable insights. Therefore, the approach enabled a careful examination of the risks and benefits of a CDST, addressing the specific issues the warning list is aimed at, namely to provide support in situations in which sedation occurs unintentionally.

There are also limitations in this study. First, we included only four palliative care pilot centres in Bavaria/Germany which limits generalisability, also given the well-known differences between services. Additionally, only few professionals participated in the online survey and answered the relevant questions. More importantly, the mixed-methods study was aimed at the whole complex intervention developed within the iSedPall project. Therefore, the warning list was only addressed in a small number of questions in the online survey. These factors significantly limit the conclusions to be drawn from the online survey regarding the warning list.

## Conclusions

This mixed-method pilot study tested the feasibility of implementation, appropriateness, and acceptability of the warning list in four specialist palliative care centres in Germany. It showed preliminary/pilot evidence that the warning list as an example of a paper-based CDST seems to be feasible to be implemented as well as appropriate and acceptable for professionals in palliative care. The warning list provides cut-off doses at which sedation is to be expected for potentially sedating drugs commonly used in palliative care. Such information is an essential basis to raise awareness of sedative (side) effects and to support professionals in their respective judgements, decision making and information giving to patients and informal carers.

In Germany, as in other countries, physicians carry the responsibility for prescribing drugs, but especially if visits of nurses take place within a home care-setting, a physician is not always available and nurses have to evaluate the situation on-site. Future research in more generalisable settings should therefore also look closer at different professional groups as well as settings, and might further explore the various types of potential use of the CDST. Another important aspect is the further exploration of how the individual needs of patients could be addressed more closely, e.g. by providing warning lists adapted to different groups of patients (e.g. according to weight, sex, substance abuse). A potential implementation in electronic form should also be explored carefully, reflecting positive and negative consequences on the daily routines and therefore patients and their professional and informal carers.

## Supplementary Information

Additional file 1. Warning list: The ‘Warning list’ contains evidence-based, expert consensus-based cut-off values for selected potentially sedating drugs commonly used in palliative care, each for a defined ‘standard patient’.

Additional file 2. Dose recommendations: The ‘Dose recommendations’ are an additional tool providing doses for the start of intentional sedation to relieve suffering.

## Data Availability

The datasets used and/or analysed during the current study are available from the corresponding author on reasonable request.
