# Supplementary material for: Clinical decision support for potentially sedating drugs in palliative care: a mixed-methods pilot study
Source: BMC Palliat Care. 2026 Oct 1;25:288. doi: 10.1186/s12904-026-02346-z (PMC13628832; doi:10.1186/s12904-026-02346-z)
Supplement: Supplementary file 1 [file 12904_2026_2346_MOESM1_ESM.pdf]

Warning list: When can sedation be expected? When to expect a sustained clinical effect?

Aim of this warning list: To raise awareness of whether sedative effects are to be expected and to support the respective judgement. Please refer to the 'Screening tool' for the two situations in which it can be used.

IMPORTANT: a) These are not dose recommendations for intentional sedation! b) The warning list cannot replace the clinical judgement regarding whether sedation is present or could occur!

Standard patient: Patient (pat.) receiving palliative care, i.e. with life-threatening illness, female or male, approx. 60 years old, body weight (bw) approx. 70 kg, no history of or current substance abuse (esp. benzodiazepines), no impairment of consciousness before the administration of the potentially sedating drug, no impairment of the blood-brain barrier, no severe impairment of kidney or liver function (GFR ≤ 30ml/min or ChildPugh B or C).

At the values indicated, a sustained clinical effect* or sedation at the degree indicated must be EXPECTED ("red flags") **for a 'standard patient'**.
CAUTION**:** These are only points of reference and cannot replace the clinical judgement in the individual case, especially taking into account all deviations from the standard patient as well as other influencing factors, including potential drug interactions and organ impairment!

Drug Group	Drug	Sustained clinical effect* to be expected (not necessarily sedation) with the stated frequency of administration	Light sedation to be expected (RASS-PAL -1 to -2)	Deep sedation to be expected (RASS-PAL -3 to -5)
Benzodiazepine	**Diazepam****	every 12-24 hours	**5 mg single dose** (independent of the route of administration)	**15 mg single dose** (independent of the route of administration)
	**Lorazepam****	every 8-12 hours	**2 mg single dose** (independent of the route of administration)	**4 mg single dose** (independent of the route of administration)
	**Midazolam**	every 2-6 hours	**2.5 mg single dose or 12 mg total daily dose s.c. or i.v.**	**5 mg single dose or 2 mg/h cont. s.c. or i.v.**
Antipsychotics	**Levomepromazine (Methotrimeprazine)**	every 12-24 hours	**12.5 mg single dose s.c. or i.v.**; 25 mg single dose p.o.	**25 mg single dose s.c. or i.v.**; 50 mg single dose p.o.
	**Haloperidol****	every 12 hours	**4 mg single dose** (independent of the route of administration)	No cut-off value, as the sedative effect is highly variable
	**Melperone****	every 6-12 hours	**50 mg single dose p.o.**	**100 mg initial dose p.o.** (i.e. without prior dose titration)
	**Pipamperone****	every 8 hours	**40 mg single dose p.o.**	No cut-off value, as there is insufficient data and clinical experience
Anaesthetic	**Propofol**	continuous administration	**0.3 mg/kg bw/h i.v.**	No cut-off value; **with each dose increase starting from 0.3 mg/kg bw/h i.v. at least -3 is to be expected**
Selective alpha-2 agonist	**Dexmedetomidine****	continuous administration	**0.4 µg/kg bw/h i.v.*** **	**0.6 µg/kg bw/h i.v.*** **
Antidepressant	**Trazodone****	every 12-24 hours	**50 mg single dose p.o.**	No cut-off value, as there is insufficient data and clinical experience

Caution: Sedation (as a side effect) is also to be anticipated for other substances, e.g. opioids!

* The "sustained clinical effect" is not limited to the sedative effect of a drug, but refers to any clinical effect. bw: bodyweight

** Drug is not recommended for intentional sedation to relieve suffering.

*** Cut-off values are based on summary of product characteristics (SmPC), not on expert consensus (due to lack of clinical experience).

CAUTION: The listed routes of administration and indications of the individual drugs deviate in part from those authorised for this drug (off-label use).

For an explanation of the selection of drugs for this list, see page 2

NOT TO BE USED FOR SEDATION

Explanation of the selection of drugs for the warning list:

The list includes potentially sedating drugs, whether or not they are recommended for intentional sedation, which

1a) are frequently used, at least partly, with the intention of sedation
OR
1b) are used for agitation/restlessness in palliative care

AND

2a) for which sufficient data are available in the literature (including the summary of product characteristics, SmPC) to allow conclusions to be drawn about the dosages at which sedative effects are to be expected
OR
2b) there is sufficient clinical experience to judge dosages with sedative effects (e.g. not the case for opioids).

Richmond Agitation-Sedation Scale modified for patients in palliative care (RASS-PAL) (Bush et al. 2014)

**4**	Combative: overtly combative, violent, immediate danger to staff (e.g. throwing items); +/- attempting to get out of bed or chair
**3**	Very agitated: pulls or removes lines (e.g. IV/SQ/Oxygen tubing) or catheter(s); aggressive, +/- attempting to get out of bed or chair
**2**	Agitated: frequent non-purposeful movement, +/- attempting to get out of bed or chair
**1**	Restless: occasional non-purposeful movement, but movements not aggressive or vigorous
**0**	Alert and calm
**-1**	Drowsy: not fully alert, but has sustained awakening (eye-opening/eye contact) to voice (10 seconds or longer)
**-2**	Light sedation: briefly awakens with eye contact to voice (less than 10 seconds)
**-3**	Moderate sedation: any movement (eye or body) or eye opening to voice (but no eye contact)
**-4**	Deep sedation: no response to voice, but any movement (eye or body) or eye opening to stimulation by light touch
**-5**	Not rousable: no response to voice or stimulation by light touch

Palliative Medicine, LMU University Hospital Munich and Palliative Medicine, University Hospital Augsburg, Germany. Adaption and translation of "Warnliste", developed in the project iSedPall, funded by the Federal Ministry of Education and Research [BMBF: 01GY2020A-C]. Version 1.0 (CC BY-SA 4.0).
Source: https://www.dgpalliativmedizin.de/isedpall/arzneimittelbezogene-entscheidungshilfen/warnliste (March 2026).
