# Supplementary material for: Clinical decision support for potentially sedating drugs in palliative care: a mixed-methods pilot study
Source: BMC Palliat Care. 2026 Oct 1;25:288. doi: 10.1186/s12904-026-02346-z (PMC13628832; doi:10.1186/s12904-026-02346-z)
Supplement: Supplementary file 2 [file 12904_2026_2346_MOESM2_ESM.pdf]

Dose recommendations for starting intentional sedation to alleviate suffering

Dose recommendations are based on the assumption of a 'standard patient'

Standard patient: Patient (pat.) receiving palliative care, i.e. with life-threatening illness, female or male, approx. 60 years old, body weight (bw) approx. 70 kg, no history of or current substance abuse (esp. benzodiazepines), no impairment of consciousness before the administration of the potentially sedating drug, no impairment of the blood-brain barrier, no severe impairment of kidney or liver function (GFR ≤ 30ml/min or ChildPugh B or C).

CAUTION: For elderly, very frail pat. or those with severe hepatic or renal impairment, start with half the stated dose if necessary, but, depending on the symptom severity, rapid dose titration may be required; higher doses may be required if there is a history of substance abuse.

SedPall best practice recommendations p. 9 (table 2)[1]:
Benzodiazepines, e.g. midazolam, are suitable for intentional sedation. Generally, these medications are the first choice, especially for patients requiring a reduction in anxiety levels and/or anti-epileptic effects. In the case of (hyperactive) delirium, they should only be administered in combination with antipsychotic medication.

	**Recommendation for starting light sedation (RASS-PAL -1 to -2)**
**Midazolam**	**0.5 – 2.5 mg** s.c. or i.v. **as an initial single dose**<br>(if administered as a bolus i.v.: 0.5 – 1.0 mg, higher dose i.v. as a slow short infusion*)<br><br>**Start with a dose 12 mg/24 h** s.c. or i.v.

SedPall best practice recommendations p. 9 (table 2)[1]:
Antipsychotics with sedating (secondary) effects, e.g. levomepromazine, are a suitable second choice medication for intentional sedation. They can be administered in combination with benzodiazepines in cases in which benzodiazepines alone are inadequate to achieve sufficient relief of suffering.

	**Recommendation for starting light sedation (RASS-PAL -1 to -2)**
**Levomepromazine** (Methotrimeprazine) **(usually in addition to midazolam)**	**12.5 mg** s.c. or i.v. **as a short infusion* as an initial single dose**<br><br>**Start with a dose 12.5 – 25 mg/24 h** s.c. or i.v.

Palliative Medicine, LMU University Hospital Munich and Palliative Medicine, University Hospital Augsburg, Germany. Adaption and translation of "Dosisempfehlungen für den Start einer gezielten Sedierung", developed in the project iSedPall, funded by the Federal Ministry of Education and Research [BMBF: 01GY2020A-C]. Version 1.0 (CC BY-SA 4.0). Source: https://www.dgpalliativmedizin.de/isedpall/arzneimittelbezogene-entscheidungshilfen/dosisempfehlungen (March 2026).

SedPall best practice recommendations p. 9 (table 2)[1]:

Propofol is suitable for intentional sedation in cases in which other types of medication have not resulted in sufficient relief of suffering. Propofol is not suitable for intentional sedation in the home care setting.

	Recommendation for starting light sedation (RASS-PAL -1 bis -2)
**Propofol**	**0.3 mg/kg bw/h** i.v.

* The term short infusion, as used in these recommendations, refers to the slow infusion of the drug solution over a period of approx. 10 – 20 minutes.

CAUTION: The methods of administration listed and indications of the individual medications deviate in part from those authorised for this medication (off-label use).

The dose recommendations for the start of "light sedation" correspond to the "cut-off values" that were agreed based on the available literature and the experience of experts in a Group Delphi workshop as the dose at which light sedation (RASS-PAL -1 to -2) can be anticipated in 'standard patients' (see above).

SedPall best practice recommendations p. 9 (table 2)[1]:

Generally, on initiation a medication dose is chosen to achieve light to moderate sedation (RASS-PAL -1 to -2). Subsequently, the dose is adjusted in accordance with the recommendation[s] [...].
SCOPE OF APPLICATION: SIPC, SPHC

In case of acute crisis (e.g. acute respiratory tract obstruction, severe haemorrhage), an initial medication dose to achieve a deep level of sedation (RASS-PAL ≤ -3) can be selected.
SCOPE OF APPLICATION: SIPC, SPHC

[1] Ostgathe C, Bausewein C, Schildmann E, Bazata J, Handtke V, Heckel M, et al. Expert-approved best practice recommendations on the use of sedative drugs and intentional sedation in specialist palliative care (SedPall). BMC Palliative Care. 2023. 22:126. https://doi.org/10.1186/s12904-023-01243-z

(*See also:* Forschungsverbund SedPall, in Kooperation mit der Dt. Gesellschaft für Palliativmedizin e.V. Handlungsempfehlung. Einsatz sedierender Medikamente in der Spezialisierten Palliativversorgung [SedPall research network, in co-operation with the German Society for Palliative Medicine e.V. Best practice recommendations. Use of sedative medication in specialised palliative care]. 2021. https://www.dgpalliativmedizin.de/images/210422_Broschu%CC%88re_SedPall_Gesamt.pdf)
